# Ovarian Germ Cell Tumors: Pictorial Essay

**DOI:** 10.3390/diagnostics12092050

**Published:** 2022-08-24

**Authors:** Nicolae Gică, Gheorghe Peltecu, Raluca Chirculescu, Corina Gică, Mihai Ciprian Stoicea, Andreea Nicoleta Serbanica, Anca Maria Panaitescu

**Affiliations:** 1 Department of Obstetrics and Gynecology, Carol Davila University of Medicine and Pharmacy, 020021 Bucharest, Romania; 2 Filantropia Clinical Hospital, 011132 Bucharest, Romania; 3 Department of Pathology, Regina Maria Health Network, 020021 Bucharest, Romania; 4 Department of Pediatrics, Fundeni Clinical Institute, Department of Pediatrics Hematology, Carol Davila University of Medicine and Pharmacy, 020021 Bucharest, Romania

**Keywords:** ovarian germ cell tumors, fertility sparing, ovarian malignancies, young women, unilateral salpingo-oophorectomy

## Abstract

Ovarian germ cell tumors of the ovary represent a histologically heterogenous group of tumors with a high incidence at reproductive age. Patients with this pathology are very often young women with amenorrhea. The aim of this article is to present a pictorial essay of this rare pathology and to promote a national tumor registry and protocol. The treatment is individualized according to age, and fertility-sparing surgery is the actual standard of surgical treatment for young patients in early stage of the disease.

## 1. Introduction

Germinal epithelium covers the surface of the infant’s ovary. Germ cell tumors derive from primitive germ cells of the embryonic ovary. They account for 20 to 30% of all ovarian malignancies [1]. Approximately 95% are benign and represented by mature cystic teratoma, while only 5% are malignant [1]. 

Ovarian malignant germ cell tumors (OMGCT) represent 2.6% of all ovarian malignancies compared to the leading position represented by epithelial ovarian malignancies (95%) [2]. It is estimated that the highest incidence of OMGCT is encountered in the first two decades of life [3].

A review study performed between 1973 and 2002 reported an incidence of OMGCTs in 3.4/1,000,000 women in the USA [3]. A survey of ovarian germ cell tumors (OGCT) in England between 1979 to 2003 found an incidence of 2.34/1,000,000 women, representing approximately 75–110 new cases per year [4].

Teilum, in 1946, first described the similarity between testicular seminoma and dysgerminoma. This similarity provided the basis for the use of common algorithms in the treatment of these tumors. With the incidence of testicular seminoma being much higher than that compared with dysgerminoma, experience gained with its treatment was significant and it was extrapolated to the treatment of dysgerminoma [5].

Germ cell tumors derive from primitive germ cells of the ovary. Teilum offered the most accepted theory about the histogenesis and interrelations of the various types of germ cell tumors [5]. Teilum considered dysgerminoma as a primitive germ cell neoplasia which did not reach the potential for further differentiation. In turn, embryonal carcinoma was considered a germ cell neoplasm containing multipotential cells able to differentiate in two different directions. If the differentiation follows the embryonal direction, the result will be the development of teratoma tumors, and if the differentiation will be toward extra-embryonal direction, the result will be the development of endodermal sinus tumors (yolk sac tumor) or choriocarcinoma (trophoblastic tumor). Making a classification in order of frequency, in the first place is dysgerminoma followed by immature teratoma, endodermal sinus tumor (yolk sac tumor), and mixed germ cell tumor. The other OMGCT, embryonal carcinoma, choriocarcinoma, and malignant struma ovarii, are less frequent. A classification of OMGCT is presented in Table 1 [6].

The most frequently used classification is that from the World Health Organization (WHO), presented in Table 2 [7].

The diagnosis of OGCT is sometimes difficult. It could be established on conventional histological specimens, but immunohistochemistry (IHC) should be used to confirm diagnosis in difficult cases. The most important IHC markers are presented in Table 3.

## 2. Clinical Presentation of GCT

Abdominal enlargement is the most common presenting symptom. Sometimes it is associated with amenorrhea and a suspicion of pregnancy is raised. Due to it having its highest incidence in the second and third decade of life, dysgerminoma is the most common ovarian tumor diagnosed during pregnancy and puerperium. Acute abdominal pain with a sudden onset could represent a complication such as torsion, hemorrhage, or rupture, and could occur in 10% of patients. When the pain is located in the right lower quadrant it could mimic an appendicitis [8].

In few cases, OGCT is associated with hormone production (human chorionic gonadotropin) and clinical signs of precocious puberty.

Diagnosis is based on complete history and clinical examination, transvaginal (when possible, or transrectal in case of virginity) or transabdominal ultrasound, and tumor markers. Serum markers recommended for the diagnosis of OGCT are lactate dehydrogenase (LDH), alpha-fetoprotein (AFP), and human chorionic gonadotropin (HCG) [9,10]. The specificity of these serum markers is presented in Table 4.

The Royal College of Obstetricians and Gynecologists (RCOG) recommends that in women under 40 years old, in suggestive circumstances for OGCT, to measure AFP, hCG, and LDH levels to diagnose this pathology [9]. The National Academy of Clinical Biochemistry, which is more specialized in tumor marker status recommends only considering AFP and hCG levels that have been extensively studied and validated by independent teams [10]. The values of these two markers are correlated with both stage of disease and survival rate in cases of OMGCT [11].

Diagnostic imaging will start with a CT scan. Abdominal and chest CT scans are recommended because OGCT could give lung metastases. Magnetic resonance imaging (MRI) could offer additional images of ovaries. In the case of a highly suspicious diagnosis, a preoperative karyotype is strongly recommended because OGCTs could arise in the dysgenetic gonads (in Turner, Klinefelter, and Swyer syndrome). If a Y chromosome is present, a dysgenetic ovary could harbor a gonadoblastoma and in more than 40% of cases this could have a malignant transformation or suffer one [12,13,14].

OMGCT follow the same staging system as epithelial ovarian and primary peritoneal cancers [15,16].

When an ovarian tumor is highly suspected to be an OGCT, surgical exploration is mandatory to achieve the histological diagnosis and staging the extent of the disease. In most of the cases surgery will be an open technique to allow the intact removal of the tumor, avoiding its rupture. Considering that surgery is performed on young women, concerned with future fertility, it should be conservative. Usually, surgery will consist of peritoneal washing, a unilateral oophorectomy, omental biopsies, and the sampling of the enlarged lymph nodes. Malignant OGCTs are mainly unilateral, but in 10–15% of cases pure dysgerminoma is bilateral. Biopsy of an apparently normal contralateral ovary is not recommended [9].

A review of the SEER data between 1988 and2006 revealed that lymph node metastases, when present, have no negative impact on the long-term outcome [17]. Thus, the guidelines do not recommend systematic lymph node dissection in OMGCT. Only enlarged nodes should be removed.

A conservative approach (intraoperative inspection of the pelvis, abdomen, and omentum, unilateral oophorectomy and lymph nodes biopsy, and excision of any visible disease) was compared to a more extensive one (oophorectomy, omentectomy, and lymph node dissection) and the results were similar in terms of recurrence and survival [18]. Thus, in the early stage of the disease, a conservative approach is recommended [19]. For cases of advanced disease (stages Ic, IIa, and greater) adjuvant chemotherapy should be recommended to preserve fertility and reduce the extent of the surgery. In cases where there is no desire for fertility, surgery will consist in total hysterectomy and bilateral salpingo-oophorectomy and additional procedures, if necessary.

## 2.1. Dysgerminoma

Ovarian dysgerminoma is the most frequent OMGCT, representing 1–2% of all ovarian primary malignancies and 32.8–37.5% of all cases of OMGCT [3,20,21]. The highest incidence is encountered in the second and third decade of life, with 80 to 85% of patients being under the age of 30. Dysgerminoma could be diagnosed during pregnancy, but its presence does not compromise tumor prognosis and the fetal outcome [22].

Dysgerminoma is often diagnosed during pregnancy or puerperium. Sometimes it is diagnosed as an emergency due to the torsion of the tumor. Although dysgerminoma is mainly located in one ovary, bilateral involvement is found in 10% of cases and this must be distinguished from metastatic dissemination.

Pure dysgerminomas do not produce hormones. It is estimated that only 5% of tumors contain syncytiotrophoblastic cells, which produce β-hCG and a positive result in a pregnancy test.

### 2.1.1. Macroscopic Features

**Figure 1:**
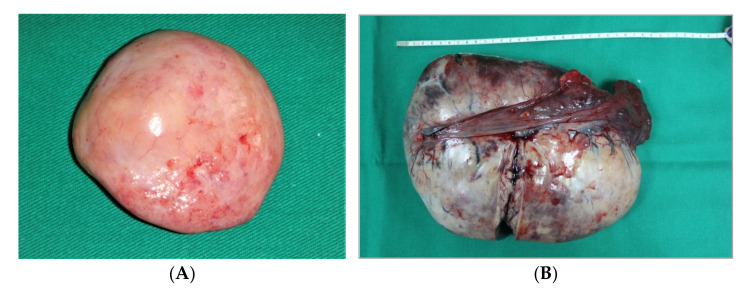
Dysgerminoma of the ovary (**A**,**B**). (Courtesy of Prof. G. Peltecu).

Dysgerminomas are well encapsulated, firm, and solid tumors, with bosselated contours, a smooth surface, and a grey-white color, and sometimes with visible vessels on their surface. The diameters vary between 5 and 15 cm (Figure 1). Rarely, large tumors could complicate with torsion and inflammatory adhesion to the surrounding structures. Hemorrhage or rupture could follow torsion.

Usually, dysgerminoma has a homogenous consistency on its cut surface, with rare exceptions when grossly visible calcifications are seen; this aspect suggests it is a gonadoblastoma.

Dysgerminoma occurs mainly unilaterally, predominantly in the right ovary (50%) than in the left ovary (33–35%), with bilaterality being rare (15–17%) [23].

**Figure 2:**
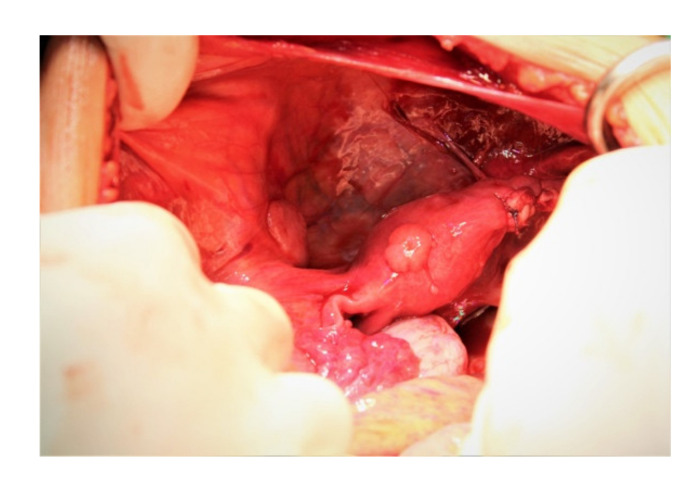
Uterine and pelvic peritoneal metastases of ovarian dysgerminoma (courtesy of Prof. G. Peltecu).

In most cases, at the time of diagnosis, the disease is confined to the ovary with no visible signs of local spreading. The most important and earliest pathway is through lymphatic spreading. In cases clinically staged as an IA disease, the incidence of microscopic metastases in the para-aortic lymph nodes is 38%. Lymphatic spreading is found firstly at the level of common iliac nodes, then in the para-aortic nodes, and finally in the mediastinal and supra-clavicular nodes. Pelvic spreading is rare (Figure 2). Visceral spreading to the liver, lungs, kidneys, and bones are late.

In 5% of cases dysgerminoma is diagnosed in patients with female phenotypes but with gonadal dysgenesis. The most frequently encountered syndromes are Turner (45, X/46, XY), Klinefelter (46, XY-male pseudo-hermaphroditism), and Swyer (46, XY-pure gonadal dysgenesis). These female patients with gonadal dysgenesis frequently develop gonadoblastomas, which are benign tumors but in 40% of cases will suffer a malignant transformation, including dysgerminoma [24]. If a dysgenetic ovary is left in situ there is a 50% risk of it developing an ovarian malignancy, firstly dysgerminoma, and the risk will increase over time. The decision to perform a bilateral oophorectomy will be based on the interest of sparing fertility or not, taken by a carefully counseled patient.

### 2.1.2. Microscopic Features

**Figure 3:**
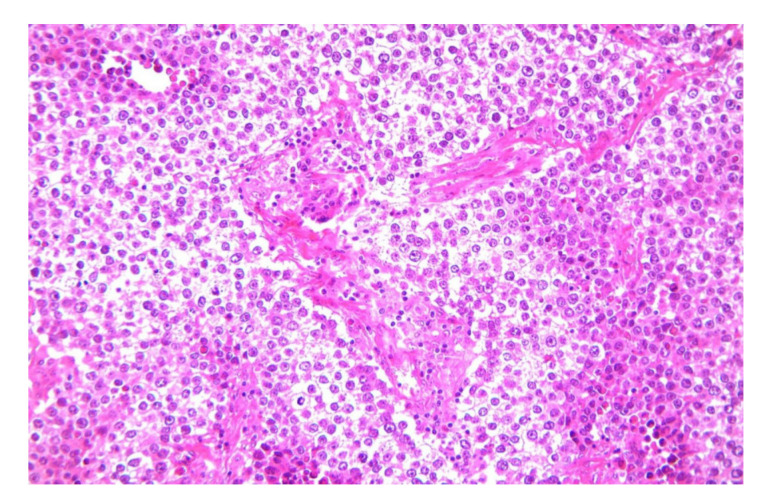
Nests of large, uniform polygonal cells with clear or eosinophilic cytoplasm, separated by fibrous septae, containing lymphocytes (20× HE) (courtesy of Dr. R. Chirculescu).

Dysgerminomas are composed of a monotonous cell population. The cells resemble primordial germ cells that grow in islands [25]. Tumor cells have a round or polygonal shape, their cytoplasm stains pale to eosinophilic and have discrete cell membranes, which are surrounded by connective tissue containing lymphocytes, mainly T-cell lymphocytes (Figure 3) [26]. The nuclei are large, rounded, and typically central or slightly eccentric, and they might contain one or a few prominent nucleoli. Some variations of the size of the cells and of the nuclei is usually seen and about 5% of dysgerminomas contain syncytiotrophoblastic giant cells, which may result in an elevated serum β-hCG level [27]. Mitotic figures are frequently seen and may vary from slight to brisk. Necrosis and hemorrhage are common [26]. Atrophic calcification is seen in some tumors in association with necrosis, hemorrhage, fibrosis, or hyaline degeneration. Sometimes microcalcifications, when present in dysgerminomas, suggest an underlying gonadoblastoma. Dysgerminoma is seen as a pure disease, nevertheless in some cases it be associated with other germ cell tumors [28]. On immunohistochemistry, the cells are strongly positive for PLAP, CD117/c-kit, OCT4, and D2-40. PLAP is not a useful marker to differentiate dysgerminoma from other malignant germ cell neoplasms since PLAP can also stain other malignant germ cell tumors, but it is a valuable marker differentiating dysgerminoma from non-germ cell malignancies. CD117/C-kit expression is not specific for dysgerminoma; it can also be seen in the solid variant of yolk sac tumors. Diffuse strong nuclear staining for OCT4 is characteristic of dysgerminoma but is also expressed in embryonal carcinoma. SALL4 is a highly sensitive marker for primitive germ cell tumors which can also show positivity in embryonal carcinoma, yolk sac tumor, and primitive areas of immature teratoma [26,29].

The differential immunohistochemical diagnoses of dysgerminoma are presented in Table 5 [30].

### 2.1.3. Treatment

Fertility-sparing surgery, which is applicable to two thirds of patients with dysgerminoma with normal karyotypes, consists of a unilateral salpingo-oophorectomy, peritoneal washing, and careful and regional lymph node inspection. Biopsy of the contralateral ovary is not routinely recommended, except is suspicion exists, because of the risk of adhesions and ovarian failures [31]. Visceral and parietal peritoneum, omentum, and diaphragm are carefully inspected, and any suspicious areas are excised and sent for biopsies. Only palpable lymph nodes are resected and sent for a pathologic examination.

In the advanced-stage disease the surgical principle applied is debulking surgery, but its application is not as aggressive as in epithelial ovarian cancers, knowing that OMGCTs have an excellent prognosis with chemotherapy.

The risk recurrence of dysgerminoma in stage IA of the disease treated only with a unilateral salpingo-oophorectomy is 15–25%, but the prognosis is good with chemotherapy, and the cure rate is almost 100%, preserving fertility in women at reproductive age. The conservative management in this category of patients, significantly reduce the anxiety and difficulties due to the potential infertility caused by bilateral salpingo-oophorectomy [32,33].

## 2.2. Mature Cystic Teratoma

A mature cystic teratoma, also called a dermoid cyst, has structures containing well-differentiated tissues derived from all the three layers, ectoderm, mesoderm, and endoderm. It is the most frequent benign ovarian tumor with the highest incidence in patients between 20 and 40 years old. Commonly it is a unilateral tumor, but in 5–10% of cases, mature cystic teratoma could be present in the contralateral ovary. Mature cystic teratoma is the most common type of GCT and they represent 15–25% of all ovarian tumors. The proportion is higher in children [34].

Most cases of MCT are diagnosed during the early reproductive life, between 30 and 35 years old [35]. They are discovered incidentally during a routine pelvic US or other imaging examination performed for other reasons, in 60% of cases. In a few cases MCTs present with non-specific signs and symptoms of an ovarian tumor.

Complications caused by MCT are rare and rarely this tumor is diagnosed by one of its complications. These could include torsion (5% of cases), rupture (2% of cases) [34], infection (1% of cases), and malignant degeneration (1.5% of cases) [36]. In cases of rupture, surgical intervention could reveal signs of a granulomatous peritoneal reaction mimicking peritoneal carcinomatosis or tuberculosis.

### 2.2.1. Ultrasound Appearance

**Figure 4:**
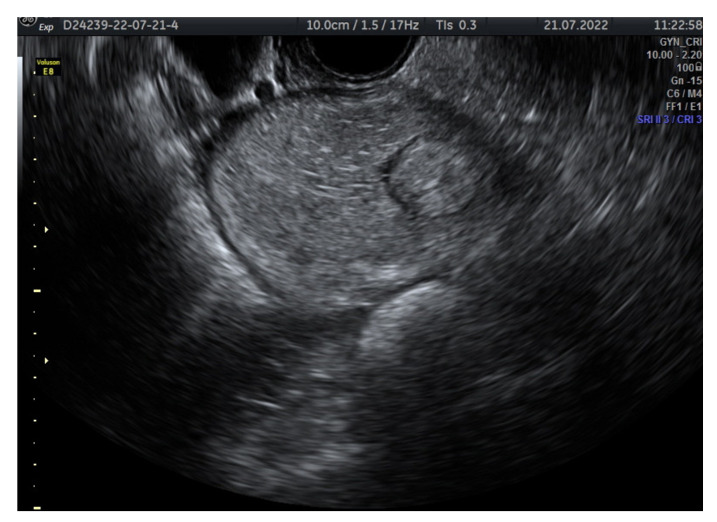
Transvaginal ultrasound of a mature teratoma. Echogenic foci or multiple hyperechoic lines are due to hairs floating.

Mature teratomas represents cystic mases, with accurate ultrasound diagnosis. This type of tumor presents some typical aspects in an ultrasound examination. The cystic masses present echogenic foci or multiple hyperechoic lines due to hairs floating. These cysts contain an echogenic shadow suggesting the presence of sebum. The echogenic masses suggest hair, teeth, or sebum (Figure 4). CT and MRI examination can easily identify the fat contained by the mature teratomas [35].

### 2.2.2. Macroscopic Appearance

**Figure 5:**
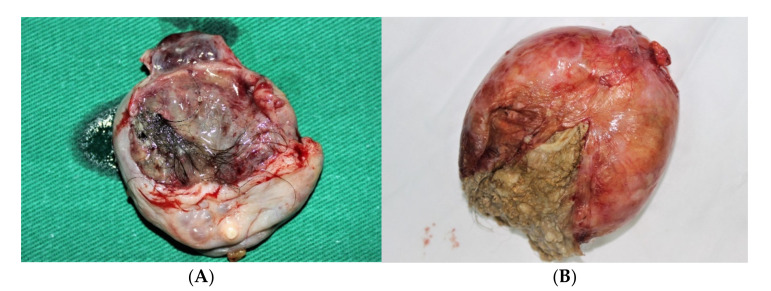
Mature cystic teratoma of the ovary (**A**,**B**). (Courtesy of Prof. G. Peltecu).

Benign teratomas are nearly always cystic tumors [29]. Usually, MCTs are round or ovoid and their average size is between 5 and 10 cm. However, their size could vary between 1 and 30 cm. MCTs have a white, smooth, thick capsule, without adhesion to the surrounding structures. The tumor is, usually, unilocular and contains sebum and hair (Figure 5). In 5–10% of cases the tumor is multilocular. The cyst wall varies in thickness, often with focal thickening or a nodular appearance. The solid nodule (Rokitansky protuberance) is composed mainly of fat tissues and is protruding into the cyst lumen [27].

Teeth are identified in 30% of cases, while bone, cartilage, adipose tissue, or brain tissue, are even rarer. Completely solid mature (benign) teratomas are very rare. Extensive solid and dense regions are unusual in a benign cystic teratoma and they should prompt careful sampling for histologic study to exclude malignancy [29].

### 2.2.3. Microscopic Features

**Figure 6:**
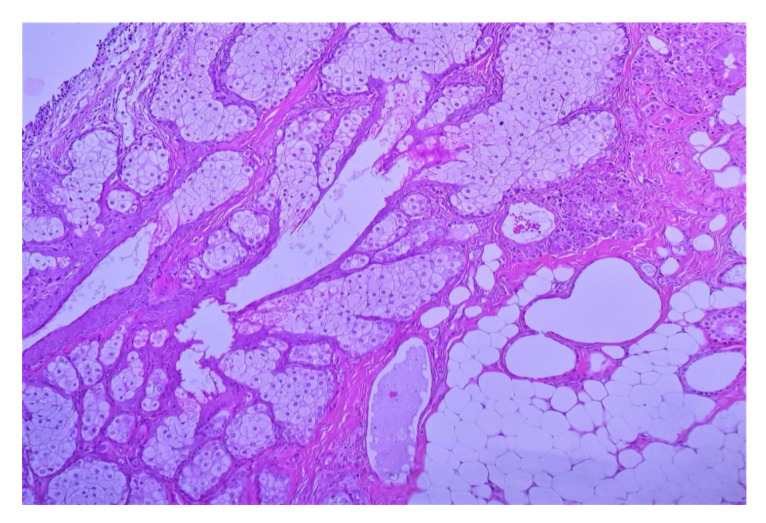
Mixture of mature, benign tissues most common squamous epithelium, and sebaceous glands; fibroadipose tissues, cartilage, or respiratory epithelium may also be found (10× HE). (Courtesy of Dr. R. Chirculescu).

**Figure 7:**
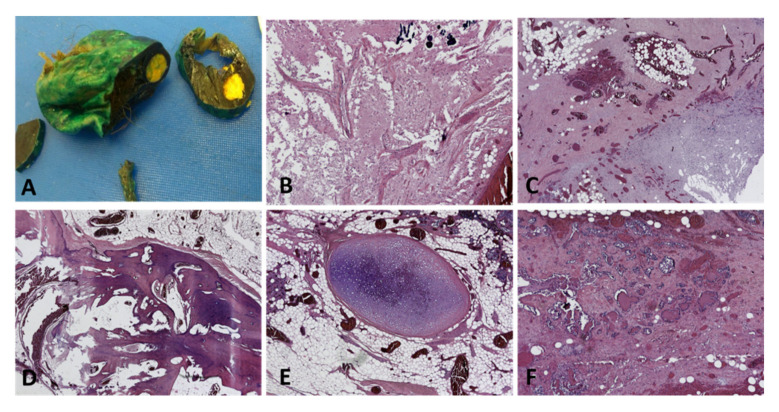
A case of a mature teratoma with torsion artefact in a 36-year-old patient: (**A**) macroscopic aspect of the right adnexa tumor; (**B**) glial type tissue (HE, ×50 magnification); (**C**) ischemic stroma with mature adipose tissue, fibroblastic areas, and thrombosed vessels (HE, ×25 magnification); (**D**) lamellar bone with scarce bone marrow, mature adipose tissue, and congestive vessels (HE, ×25 magnification); (**E**) mature cartilage and adipose tissue with congestive and thrombosed vessels (HE, ×25 magnification); (**F**) ischemic stroma with mature adipose tissue, thyroid follicles, and respiratory type glands (HE, ×50 magnification). (Courtesy of Dr. M. Stoicea).

Mature teratomas are made of a variable mixture of ectodermal, mesodermal, and endodermal tissues [29]. Ectodermal elements predominate in almost all cases, particularly epidermis and pilosebaceous structures. The outer layer of the wall of the cyst is composed of ovarian stroma, while the inner side of the cyst is lined mainly by keratinized squamous epithelium and usually contains abundant sebaceous and sweat glands associated with adipose tissues (Figure 6). Hair and hair follicles are usually present. Occasionally, the cyst wall may be lined with ciliated respiratory mucosa or gastrointestinal epithelium. Sometimes the lining epithelium is commonly eroded resulting in the escape of the cyst contents into the surrounding tissues, and this may be associated with a foreign body giant cell reaction. Other ectodermal derivatives are brain tissue, glia, neural tissue, retina, choroid plexus, or ganglia. The most common mesodermal tissues are smooth or striated muscles, adipose tissues, bones, teeth, and cartilage. The most common endodermal tissues are digestive tract tissues, including esophageal squamous epithelium, gastric epithelium, and intestinal epithelium with or without endocrine cells, respiratory epithelium, and thyroid tissues (Figure 7). All these tissues may be scattered diffusely, usually haphazardly organized within the tumor [26].

### 2.2.4. Treatment

MCT of the ovary has a surgical treatment represented by a simple cystectomy. Rarely unilateral adnexectomy is applied, especially in very large cysts or in the menopause. The MCT should be completely removed, and pathology examinations should be carefully performed to exclude the immature elements. Laparotomy or laparoscopy could be both, recommended. The choice between laparotomy and laparoscopy is determined by the size of the tumor. There are studies showing an increased rate of spillage related to laparoscopy (18% vs. 1%). Other additional risks are the greater operating time and recurrence, more frequent associated with laparoscopy [37]. Spillage is associated with the risk of developing chemical peritonitis (0.2% of cases) and adhesion.

## 2.3. Yolk Sac Tumor (Endodermal Sinus Tumor)

The term yolk sac tumor (YST) replaces the old term of endodermal sinus tumor. By incidence it represents 1% of all ovarian malignancies and 14.5% to 16.4% of all OMGCTs. The YST is the third most frequent OMGCT. It is a rare tumor, its incidence being half of that of dysgerminoma. Although the age of the distribution is wide, between infancy and late menopause, the highest incidence is at around 30 years of age, 18 being the median age [22,38]. It is a highly malignant tumor that had a very poor prognosis in the past. Nowadays, due to chemotherapy, its prognosis is very much improved allowing for fertility-sparing surgery and the hope for childbearing. The yolk sac tumor has a very rapid grow. Kurman et al., in a study collecting 71 cases, described two patients with ovarian YSTs of 9 and 12 cm at the time of diagnosis, who had normal findings at pelvic examinations one month before [26]. Another example of rapid growth is that of a pregnant woman with normal findings at the first US confirming a pregnancy which was discovered to be a 23 cm tumor at 14 weeks’ gestation. The rapid growth could be explained by the high rate of capsular rupture seen during surgery. The most relevant clinical signs and symptoms are abdominal distension and pain. Due to the rapid growth of the evolution of tumor, it could be complicated by torsion or rupture. These complications are reported through the occurrence of acute pain and clinical signs of an acute abdomen. During the fetal life, AFP is produced by a yolk sac, but also by the liver and upper gastro-intestinal tract. Yolk sac tumors produce AFP, and even if it is not pathognomonic, its serum levels are useful for the diagnosis of a primary ovarian malignancy, its metastases or its recurrence. The fall of AFP to normal values in the postoperative period is a good prognostic sign, while its increase rises the suspicion of the onset of recurrence, often earlier than other clinical or imaging signs can [39].

### 2.3.1. Macroscopic Features

**Figure 8:**
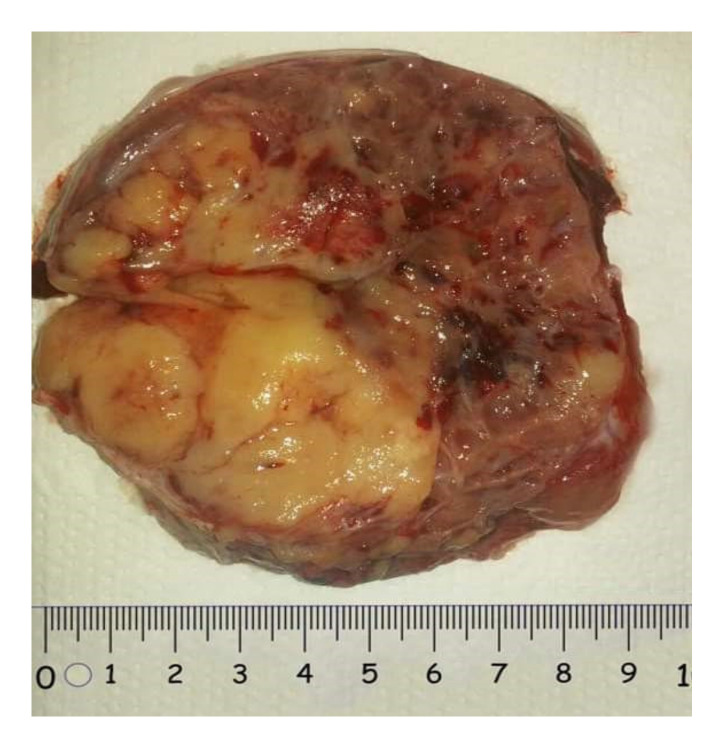
Yolk sac tumor (courtesy of Prof. G. Peltecu).

Yolk sacs tumors are in most of the cases unilateral, with a predilection for the right ovary. The iinvolvement of both ovaries is typically a manifestation of metastatic spread. The tumor is usually large, varying from 3 to 30 cm in diameter, with an average diameter of 15 cm. The tumor is usually oval-shaped, encapsulated, solid and cystic, fleshy with frequent necrosis and hemorrhage (Figure 8) [26]. If calcifications are found, they should raise the suspicion of another germ cell tumor component. It is not unusual to find a mature cystic teratoma in the contralateral ovary [25].

### 2.3.2. Microscopic Features

Characteristically, yolk sac tumors have an admixture of different patterns. The most common pattern is the microcystic one. Other tumoral architectural patterns are macrocystic, myxomatous, solid, endodermal sinus, polyvesicular, alveolar-glandular, papillary, hepatoid, and glandular. The microcystic or reticular pattern and the myxomatous pattern is composed of a loose, poorly formed network of channels lined by flat, mesothelial-like cells with pleomorphic, hyperchromatic nuclei. The macrocystic pattern is similar to the microcystic pattern, but with larger cysts.

**Figure 9:**
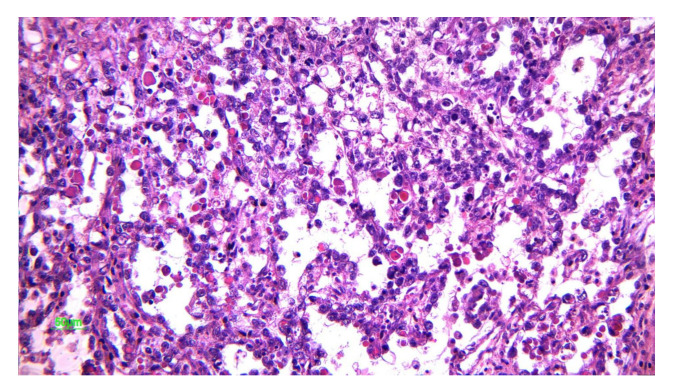
Anastomosing channels and variably sized cysts lined by neoplastic cells with clear and eosinophilic cytoplasm—eosinophilic hyaline droplets in the cysts (20× HE). (Courtesy of Dr. R. Chirculescu).

The endodermal sinus pattern contains the classic Schiller-Duval body, consisting of a narrow band of connective tissue with a capillary in the center. The cells are cuboidal, or low columnar, epithelial-like cells with large and prominent nucleoli and show mitotic activity. The presence of these structures is diagnostic of a yolk sac tumor; however, their absence does not exclude the diagnosis as only one-third of cases have Schiller–Duval bodies. The polyvesicular vitelline pattern is composed of numerous cysts and vesicles lined by flat or columnar epithelium, surrounded by a compact connective tissue stroma. Hyaline droplets may be present either within the tumor cells or outside them (Figure 9). The droplets may be observed in tumors with any of the histologic patterns described, and their identification is a helpful diagnostic feature. Nevertheless, their presence is not diagnostic of YST, often poorly differentiated neoplasms, and particularly clear cell carcinoma can contain hyaline droplets [25,26,40]. The alveolar-glandular pattern contains variable amounts of gland-like or alveoli-like spaces structures lined by flat, cuboidal, or columnar tumor cells, surrounded by a myxoid stroma [40]. The hepatoid pattern is present in up to 40% of yolk sac tumors; this pattern is composed of nests and cords of polygonal eosinophilic cells with round nuclei with eosinophilic, uniform, or granular cytoplasm, showing a solid pattern. This pattern has a remarkable resemblance to hepatocytes [26].

**Figure 10:**
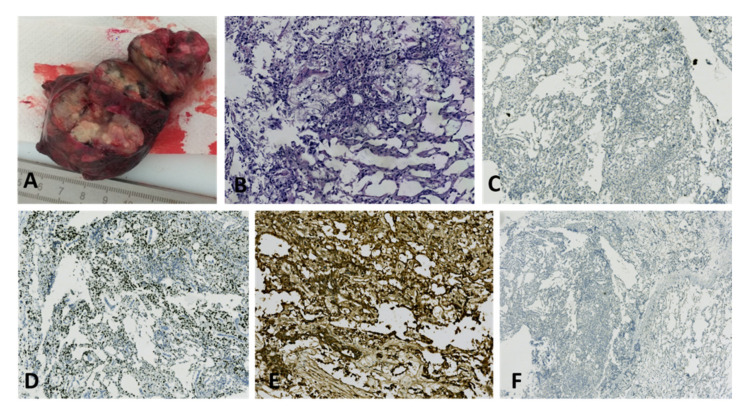
Yolk sac tumor in a 67-year-old patient: (**A**) Macroscopic aspect of a right ovarian tumor with extensive necrosis and hemorrhage; (**B**) High nuclear grade neoplasia with reticular architecture and microcysts (HE, ×100 magnification); (**C**) No immunohistochemical reaction for CD30 in tumor cells (×50 magnification); (**D**) Diffuse immunohistochemical reactivity for Sall4 in tumor cells (×50 magnification); (**E**) Diffuse intense immunohistochemical reactivity for AFP in tumor cells (×100 magnification); (**F**) No immunohistochemical reaction for OCT4 in tumor cells (×25 magnification). (Courtesy of Dr. M. Stoicea).

Immunohistochemistry (IHC) of YST is usually positive for AFP, however its sensitivity is relatively low. Other positive markers are glypican-3, SALL4, and LIN28. HepPar-1 is positive in hepatoid YST, but this marker is not helpful in the differential diagnosis of other tumors with hepatoid differentiation. Yolk sac tumors are usually negative for ER, PR, EMA, PAX8, CK7, OCT4, D2-40, and SOX2 (25). Also, CD30 should be negative (Figure 10) [30].

**Figure 11:**
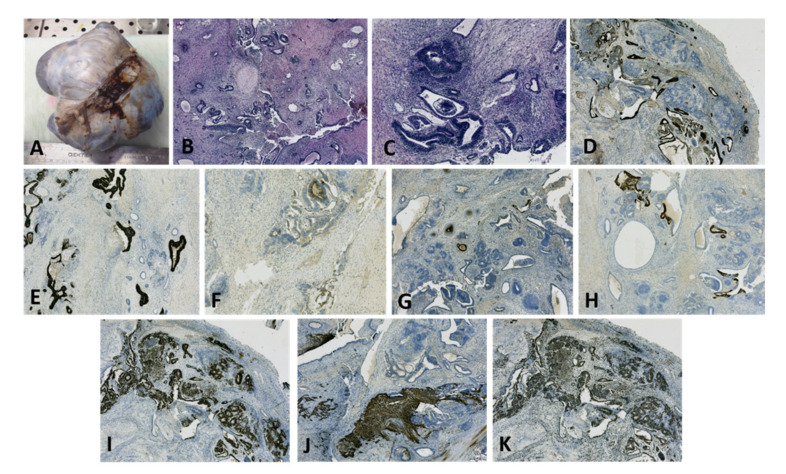
Polycystic right ovarian tumor with composite areas, microscopically diagnosed as malignant mixed germinal cell tumor (embryonal carcinoma, immature teratoma with neuroectodermal differentiation, and minor yolk sac component) in a 33-year-old patient: (**A**) Macroscopic appearance of a large ovarian mass; (**B**) Mixed histopathological pattern malignancy consisting of predominantly irregular, angulated glandular structures, rare Schiller-Duval bodies, and atypical glial tissue (HE, ×25 magnification); (**C**) Composite tumor including irregular, angulated glandular structures, and immature teratoma with neuroectodermal tubules (HE, ×50 magnification); (**D**) AE1/AE3 immunoreactivity in epithelial cells; no reaction in neuroectodermal tubules (×25 magnification); (**E**) CD30 immunoreactivity in epithelial cells; no reaction in neuroectodermal tubules and yolk sac elements (×25 magnification); (**F**) Glypican-3 immunoreactivity in yolk sac elements (×50 magnification); (**G**) AFP immunoreactivity in yolk sac elements (×25 magnification); (**H**) OCT4 immunoreactivity in embryonal carcinoma elements (×25 magnification); (**I**) Sall4 immunoreactivity in neuroectodermal tubules and rosettes (×25 magnification); (**J**) GFAP immunoreactivity in immature neuroectodermal structures (×25 magnification); (**K**) high proliferation index (Ki67, ×25 magnification). (Courtesy of Dr. M. Stoicea).

Yolk sac tumor components can be encountered in combination with other types of germ cell malignancies, including extra-gestational choriocarcinoma, embryonal carcinoma, and immature teratoma. The most frequent presentation is dysgerminoma with a yolk sac tumor [41]. These particular presentations are classified as mixed germ cell tumors; they represent approximately 10% of germ cell malignancies and can metastasize with different tumor morphologies. For therapeutic purposes, the exact extent of each component should be stated in the pathology report. Extensive sampling and an immunohistochemical analysis play a very important role in the assessment of mixed germ cell tumors (Figure 11) [42].

### 2.3.3. Treatment

The management of a YST is not based on randomized clinical trials because of the limited number of cases reported. Due to its rapid growth, YST is diagnosed early in 70% of cases when the tumor is apparently confined to one ovary. This intraoperative evaluation corresponds to stage IA of FIGO, but usually there are occult metastases. In 30% of cases the disease is extended intraperitoneally by the time of diagnosis. Surgery consists of a laparotomy followed by intraoperative staging. In stage IA-IB, for patients who wish to preserve fertility, surgery should consist in a unilateral salpingo-oophorectomy, peritoneal washing, omentectomy, and multiple peritoneal biopsies of any suspicious lesions [43].

For stages IC to IV, debulking surgery is recommended. In very carefully selected cases, fertility-sparing surgery could be offered, followed by three to four cycles of BEP chemotherapy (Bleomycin, Etoposide, Cisplatin) [44].

The risk of metastases is high and occur early on, the main sites being the retroperitoneal lymph nodes, the liver, the lungs, and the bowel. Chemotherapy should be offered in stages IA-IB, with fertility-sparing surgery if postoperative tumor markers are positive [44]. After chemotherapy there is a standard follow-up. The 5-year survival rate is very good for stages I and II (95% and 90%), and poor for stages III and IV (30% and 25%) [43,45].

## 2.4. Immature Teratoma

Immature teratomas (IT) represent 35.6–36.2% of all OMGCTs, taking the second place after dysgerminoma. Pure ITs represent only 1% of all ovarian teratomas and their peak incidence is between 15 and 19 years of age. It is a highly aggressive malignancy, being responsible for 30% of death from ovarian cancer in women under the age of 20 [3,21]. Most of them contain tissues derived from the ectoderm, mesoderm, and endoderm. A limited number are composed, predominantly or exclusively, by monodermal tissues. In contrast to mature cystic teratoma, ITs contains immature or embryonic tissues. It is considered that the presence of immature embryonic structures within an ovarian tumor it is the sign of an IT.

An immature teratoma is much larger than a mature cystic teratoma (size ranges from 14–25 cm to 7 cm) [46,47]. An immature teratoma is a solid tumor or has a mixed structure containing small cysts. These cysts contain serous or mucinous fluid or fatty sebaceous content [48]. They have a grey-tan color and a fleshy consistency. The surface of an IT has a variegated aspect due to hemorrhage and necrosis.

In 25% of cases, ovarian ITs may be associated with peritoneal spread that could, secondary to adjuvant chemotherapy, turn into mature implants (growing teratoma syndrome) and/or gliomatosis peritonei (GP), described histologically as the presence of pure mature glial tissues [49]. Rarely, gliomatosis peritonei is associated with the presence of mature glial tissue in the pelvic lymph nodes. Gliomatosis peritonei has a clinical benign evolution and the presence of lymph nodes gliomatosis does not represent an indication for chemotherapy [50].

Immature teratoma are classified according to a histological grading system based on the number of microscopic low-power fields containing immature neuroectodermal tissue present on any one slide (Table 6) [51].

Recently, the initial system of histology grading was changed to a simpler one, describing the LG (low grade, corresponding to classical grading 1) and the HG (high grade, corresponding to grading 2 and 3). The computing tomography (CT) imaging reveals large solid-cystic tumors that are ovular, or irregular in shape, well or ill defined, with an average size of 9.7 cm, with a hemorrhage inside the tumor, large vessels, and a capsular rupture. Very rarely, a CT can identify intratumor calcification and fatty tissue, and these are signs of YSTs containing teratoma structures [52].

Although not specific, a high proportion of patients with YST, either pure or mixed forms, have a significant high level of AFP.

### 2.4.1. Macroscopic Features

Most tumors are predominantly solid, but the gross findings vary according to the degree of tissue differentiation [40]. The tumor is usually unilateral, large, ranging from 6 to 42 cm in diameter with average of 18.5 cm. It can be round, ovoid, or lobulated, sometimes with an irregular or ruptured external surface. On the cut section, the solid areas are gray-tan to pink, soft, and fleshy, with frequent areas of necrosis and hemorrhage. Cystic structures can be present, usually filled with serous or mucinous fluid or a fatty material. The tumor can contain areas resembling normal tissues such as bone or cartilage elements and in up to 26% of cases, a dermoid cyst may be grossly identified within the immature teratoma or a mature cystic teratoma may coexist in the opposite ovary [25,26].

### 2.4.2. Microscopic Features

Most of the tumors consist of a variable admixture of mature and immature elements, in various stages of immaturity. Immature elements can originate in any of the three embryonic layers. The most common immature element is neuroepithelium, of neuroectodermal origin, which is the easiest immature tissue to recognize and quantify for the purpose of grading [29]. The neuroepithelium is composed predominantly of small, blue neuroblasts, primitive neuroepithelial rosettes, and tubules lined by columnar cells with stratified hyperchromatic nuclei with a high nuclear-to-cytoplasmic (N:C) ratio, and with numerous mitotic figures and apoptotic bodies. The amount of the primitive neuroepithelial component that is included is an important factor in grading and determining the prognosis of the tumor. The presence of other immature components, such as immature cartilage, bone, skeletal muscle, and glandular structures or other embryonic elements, is not sufficient for the diagnosis of an immature teratoma [28]. Endodermal tissues are usually less extensive than ectodermal or mesodermal tissues in an IT. Occasionally, ITs can be seen to be admixed with other types of germ cell tumor, commonly YSTs.

The grading system of ITs rates them from grade one to grade three and this is based on the relative quantities of the immature neural tissue. An alternative two-grade system has been proposed, but it is not widely used [29].

Immunohistochemistry (IHC) does not play a major part in the diagnosis of an IT. In most cases, the simple examination of the hematoxylin and eosin-stained slides establish the diagnostic. However, in cases of mixed germinal tumors, IHC can assisst in separating immature neuroectodermal islands from other intricated malignant components, especially embryonal carcinoma [42]. Immature neuroectodermal derivatives express Sall4a, SOX2, and glypican-3 [53]. Other markers such as GFAP sau S100 can be used to highlight other neural components such as glial cells or neuroblasts [30].

**Figure 12:**
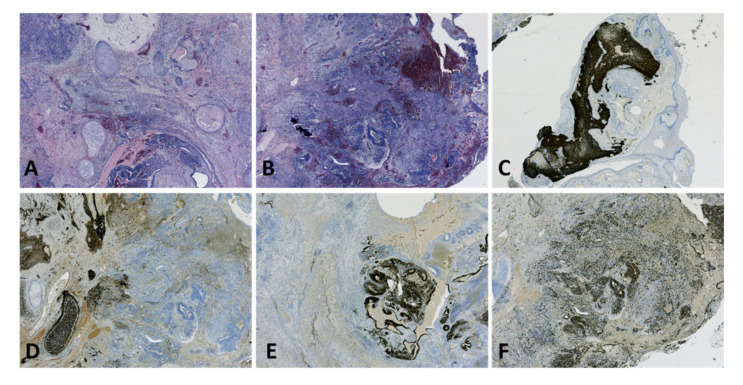
Immature teratoma with neuroectodermal differentiation: (**A**) Teratoid lesion composed of variably dilated glandular lumina with respiratory and digestive type epithelium and bland cartilage islands; immature neural tissue (lower part of the image) (HE, ×25 magnification); (**B**) Extensive (35%) areas of immature neural structures (neuroectodermal tubules and rosettes): immature teratoma with neuroectodermal differentiation, high grade (G3) (HE, ×25 magnification); (**C**) GFAP immunohistochemical staining in immature neuroectodermal areas, a lack of reaction in mature teratoid elements (×25 magnification); (**D**) S100 immunohistochemical staining in mature neural structures and cartilage islands, a weak focal reaction in immature neuroectodermal areas; lack of reaction in mature teratoid elements (×25 magnification); (**E**) Sall4 immunohistochemical staining in immature neuroectodermal areas, a lack of reaction in mature teratoid elements (×25 magnification); (**F**) Increased mitotic activity in immature neuroectodermal areas (Ki67, ×25 magnification). (Courtesy of Dr. M. Stoicea).

Neuroectodermal elements can be highlighted by the glial fibrillary acid protein (GFAP), synaptophysin, S100, CD99, and NSE. GFRalpha-1 can be used for the identification of immature neuroepithelium, but it is not widely available in many laboratories (Figure 12) [28].

### 2.4.3. Treatment

The prognosis of an IT is influenced by the histologic grade in its early stages and the presence of metastases in advanced stages. These two risk factors have different significations. The grade of the primary tumor, which is based on the degree of immaturity of the neuroepithelial tissue, is corelated with the probability of extraovarian spreading; while the presence of metastases is corelated with the possibility of recurrence and often fatal outcome. Other risk factors are the tumor size, spontaneous or intraoperative rupture of the tumor, and older age. Recurrences occur commonly within the first year.

There is a consensus that the fertility-sparing surgery (unilateral salpingo-oophorectomy) should be offered to young patients, with a tumors stage FIGO I or II (grade one or two). Spontaneous tumor rupture during surgery is associated with the risk of the dissemination of disease. The evaluation of the contralateral ovary should be carefully performed because this could harbor a dermoid cyst.

For patients with a FIGO stage I or II (grade three of immaturity), and patients in stage FIGO III (any grade of maturity), or in case of recurrences, chemotherapy should be recommended. Modern chemotherapy has long-term excellent results, with an overall disease-free survival rate of 95% [54,55].

In cases of second-look surgery for recurrences post chemotherapy, all the metastatic tumors excised must be carefully examined, histologically, to know their degree of maturation. If they are all mature (peritoneal gliomatosis), then the prognosis is surprisingly good. Radiation therapy does not have a place in the treatment of IT.

## 2.5. Embryonal Carcinoma

Embryonal carcinoma (EC) is a rare OMGCT, while its homologous counterpart is relatively common in the testis. In most of the cases EC is a constituent part of it is a mixed OGCT containing especially a YST or other tumor types. Pure ECs are extremely rare in the ovaries. The reason why there is such a discrepancy between the ovary and the testis is not known [56].

There are case reports where ECs developed from pre-existing gonadoblastomas. The EC is the most undifferentiated OGCT. It has the capacity to differentiate towards embryonic (teratoma) or extraembryonic structures (YST). It is difficult for the classical hematoxylin-eosin staining to establish the diagnosis so IHC is helpful to confirm it and clearly separate the limits between ECs and YSTs.

Embryonal carcinoma is the most frequently encountered during the second and third decade of life, within a median age of 15, but there are case reports mentioning women older than 50 years of age [57].

The most common clinical sign is abdominal enlargement. In contrast to other OGCTs, like dysgerminoma or YSTs, an EC is frequently accompanied by signs of hormonal activity such as uterine bleeding or precocious pseudo-puberty. Among the serum markers, β-hCG has frequently high levels.

The imaging investigations (US, CT, and MRI) are not specific in establishing the diagnosis, and this is similar to other OGCTs, but serum markers (β-hCG and AFP) are useful.

### 2.5.1. Macroscopic Features

**Figure 13:**
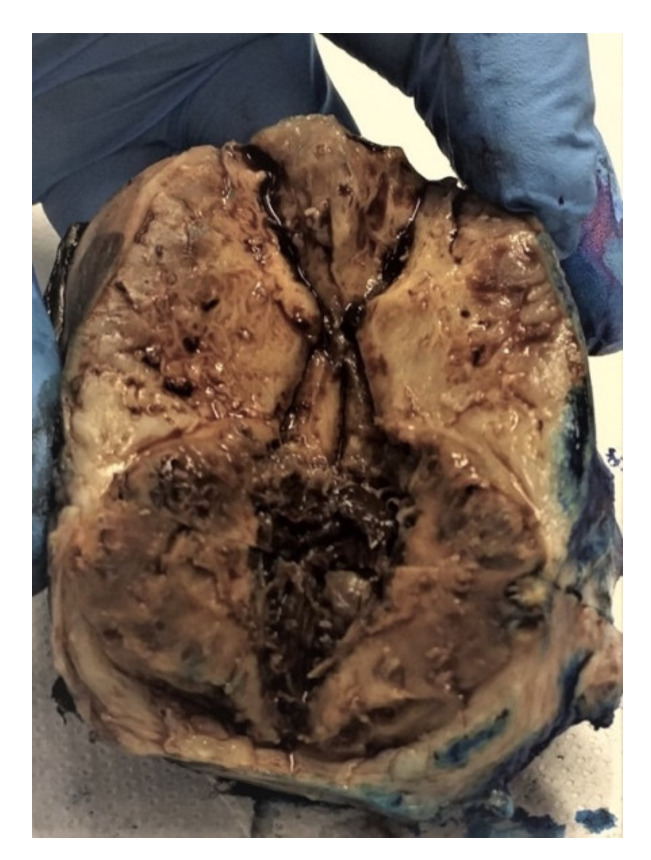
Encapsulated ovarian tumor diagnosed as embryonal carcinoma (courtesy of Dr. M. Stoicea).

Because an EC is usually a component of mixed germ cell tumors, the gross appearance can vary due to the different components present within the tumor. These tumors are typically unilateral and large, with an average diameter of 17 cm, and smooth and glistening surface. The cut surface is soft and fleshy with alternating gray, tan, and yellow colors and have cysts with mucous material. Foci of necrosis and hemorrhage are frequent, especially if the tumor is large (Figure 13) [26].

### 2.5.2. Microscopic Features

An EC is composed of solid aggregates of epithelioid, large, polygonal, or ovoid cells with vesicular nuclei that contain coarse basophilic chromatin and one or more prominent nucleoli and numerous mitotic figures. The cell membranes are well defined, and the cytoplasm is abundant and usually amphophilic or clear. The tumor can form gland-like spaces and papillae, often with central necrosis, is often admixed with solid areas. Syncytiotrophoblasts and/or intermediate trophoblasts are present in most cases, usually located at the periphery of the tumor or within the hemorrhagic areas, but cytotrophoblasts are absent, except in true mixed germ cell tumors with choriocarcinoma. Necrosis is common, either as focal confluent foci or as single cell necrosis. Eosinophilic droplets, as seen in yolk sac tumors, can be present. Whenever embryonal carcinoma is identified in an ovarian tumor it is mandatory to search for other germ cell tumor types, as an EC is the only exceptional, pure tumor [26,29].

Embryonal carcinoma should be confirmed immunohistochemically. Differentiating ECs from others germ cell tumors is important because of their different prognoses and responses to treatment. Embryonal carcinoma is strongly and diffusely positive for cytokeratin, PLAP, CD30, SALL4, OCT4, and SOX2. EMA is almost always negative and only occasionally, the tumor is positive for AFP and negative for glypican and CD117. A panel including CD117, SOX2, and CD30 can distinguish between an EC and dysgerminoma, and a panel including AFP, glypican-3, OCT4, and CD30 can distinguish between an EC and a yolk sac tumor [30,40].

### 2.5.3. Treatment

Embryonal carcinoma has a highly malignant potential. Its aggressiveness is mainly local, and it spreads throughout the abdominal cavity. The distant metastases develop late, and lung, liver, and retroperitoneal lymph nodes are the predilect sites for their presence.

At the time of surgical diagnosis about 60% of the patients are in stage IA FIGO. If it were based only on conservative surgery (unilateral salpingo-oophorectomy), the prognosis would be poor because of the occult metastases already present at the time of diagnosis. Surgical treatment in early stage must be followed by adjuvant chemotherapy (CMT). For advanced cases it is recommended that cytoreductive surgery is followed by CMT. Radiotherapy is not recommended as an adjuvant therapy for an EC. The follow-up is performed with serum markers, β-hCG and AFP.

## 2.6. Monodermal, Specialized, and Somatic Malignancies in Teratomas

Monodermal teratomas are usually benign cystic lesions consisting of terminally differentiated derivatives from a single germ layer that is either ectodermal or endodermal. This category of germ cell neoplasia includes epidermoid cysts, neuroectodermal cysts, rarely respiratory type cysts, prolactinomas, or corticotroph adenomas. This category of lesions excludes struma ovarii, which are considered a specialized type of teratoma [58].

Although composed of highly differentiated types of cells, monodermal teratomas can generate malignant tumors, as seen in teratomas with a double or triple germ layer origin. These malignant counterparts are grouped as a distinct category [59].

**Figure 14:**
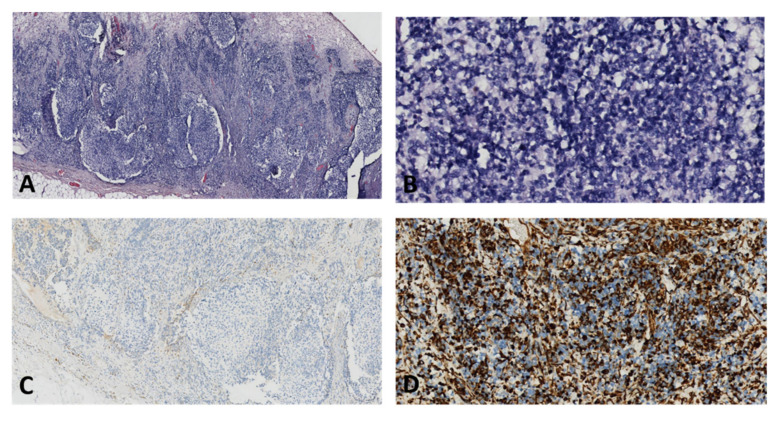

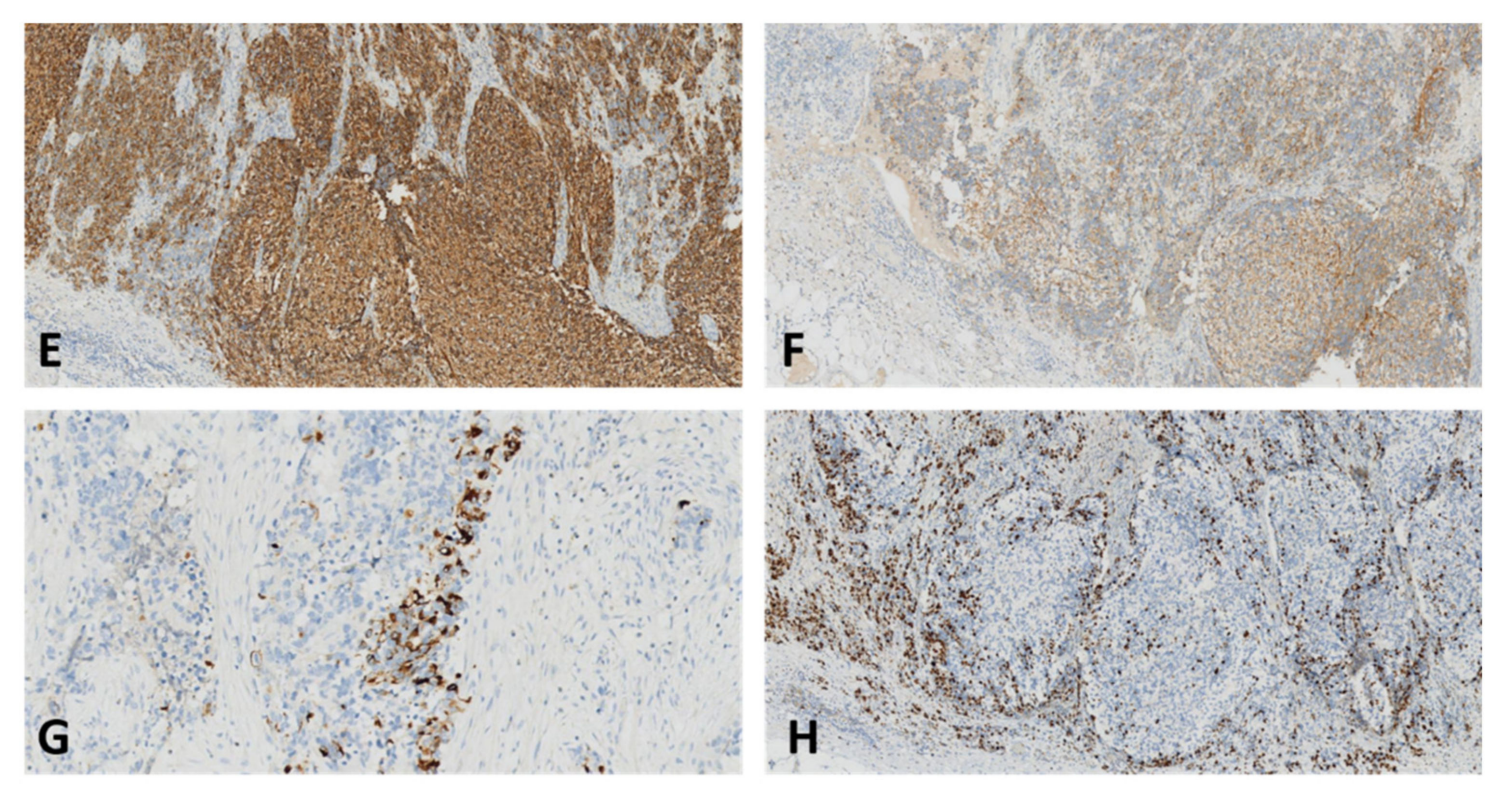
Teratomas with malignant transformation, medulloblastoma type: (**A**) Poorly differentiated small cell proliferation with microinsular and diffuse pattern (HE, ×20 magnification); (**B**) Poorly differentiated small cell proliferation with hyperchromatic nuclei (HE, ×170 magnification); (**C**) EpCAM negative (×40 magnification); (**D**) Vimentin positivity in tumor cells (×100 magnification); (**E**) CD56 positivity in tumor cells (×40 magnification); (**F**) Synaptophysin positivity in tumor cells (×20 magnification); (**G**) Focal-GFAP positivity in tumor cells (×100 magnification); (**H**) Proliferation index with marginal zonation (Ki67, ×40 magnification). (Courtesy of Dr. M. Stoicea).

Rarely, ovarian teratoid tumors contain neuroectodermal derivatives with various degrees of differentiation and pathological appearances, including ependymomas, ependymoblastomas, astrocytomas, glioblastomas, oligodendrogliomas, medulloblastomas, neurocytomas, and neuroblastomas (Figure 14) [60].

## 2.7. Struma Ovarii

A struma ovarii tumor (SOT) is a subtype of ovarian teratoma composed, predominantly or entirely, of thyroid tissue and containing large and small follicles filled with colloid material. They represent 0.3–1% of all mature cystic teratomas [61]. Commonly, 5–13% of all mature cystic teratomas of the ovary contain areas of thyroid tissue. Establishing the diagnosis of an SOT requires that more than 50% of the tumor is made of thyroid tissue or the thyroid tissue is functionally active [62].

The incidence of SOTs related to age is the same with mature cystic teratomas, with most of the cases occurring in the third and fourth decade of life. There are no specific symptoms or signs in most of the cases. In some cases, an SOT is found incidentally during an US, together with ascites in 30% of cases, but this does not suggest malignancy [63]. Hydrothorax was mentioned in some case reports, suggesting an initial diagnosis of Meigs syndrome. Clinical signs of thyrotoxicosis are rarely encountered (in less than 5% of cases) [64].

Imaging investigations (US, CT, and MRI) are not specific, overlapping with other OGCTs, and they present as a complex mass with cystic and solid areas [61].

### 2.7.1. Macroscopic Features

**Figure 15:**
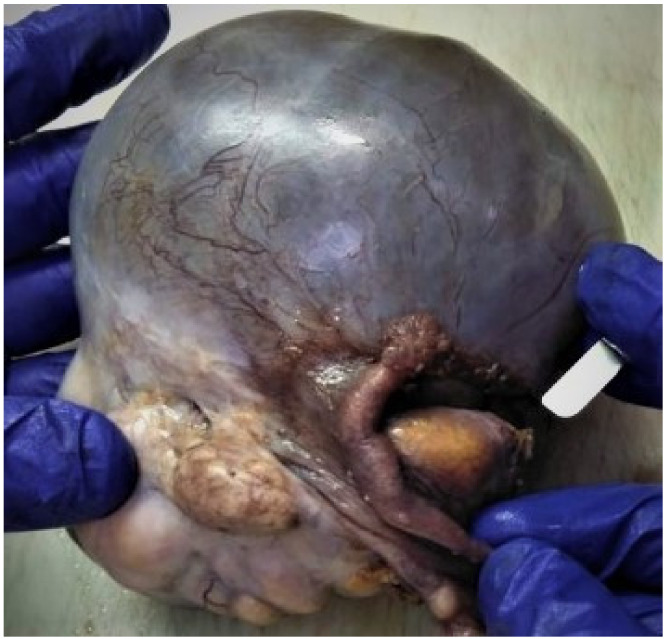
Struma ovarii (courtesy of Dr. M. Stoicea).

A struma ovarii is usually a unilateral tumor and it ranges in size from a microscopic neoplasm to a large solid or cystic mass. The average size is 5–10 cm. The struma may be pure, but it is more commonly associated with another tumor, usually a dermoid cyst. Grossly, the thyroid component has the characteristic appearance of a goiter, with a smooth or nodular outer surface. The tumor is soft and gelatinous, with a brown to green cut surface. Cystic struma ovarii can be multiloculated or, rarely, uniloculated [26,28] (Figure 15).

### 2.7.2. Microscopic Features

**Figure 16:**
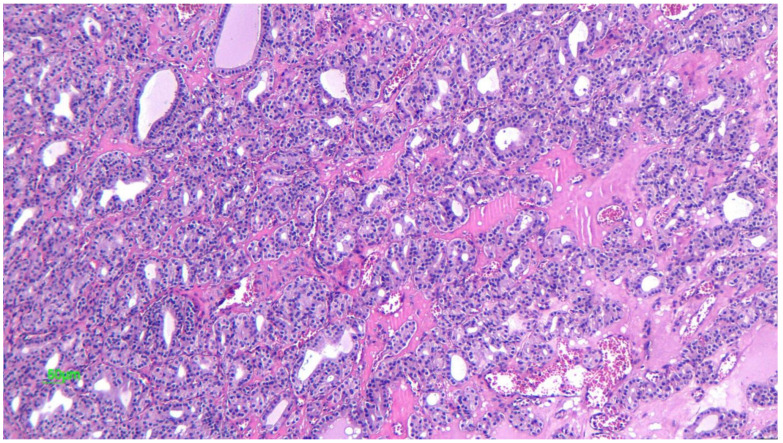
Macro- and microfollicles containing little or no colloid (10× HE) (courtesy of Dr R. Chirculescu).

A struma ovarii is composed of mature thyroid tissues consisting of follicles lined by cuboidal or columnar epithelial cells with uniform round nuclei and moderate amounts of eosinophilic cytoplasm. The follicles contain an eosinophilic, PAS-positive colloid, which may contain birefringent calcium oxalate crystals. There may be considerable variation in the size of the follicles—large, with a large amount of colloid, or small without colloid (Figure 16). Parafollicular cells (C-cells) are absent. The nuclei are typically round- or oval-shaped with minimal cytologic atypia, and rare mitotic figures. In general, these tumors have scanted intervening stroma, and features of thyroid goiters such as a hemorrhage and a cystic change can be present. The tumors can be associated with other tumors such as a dermoid cyst, a mucinous tumor, or a Brenner tumor.

**Figure 17:**
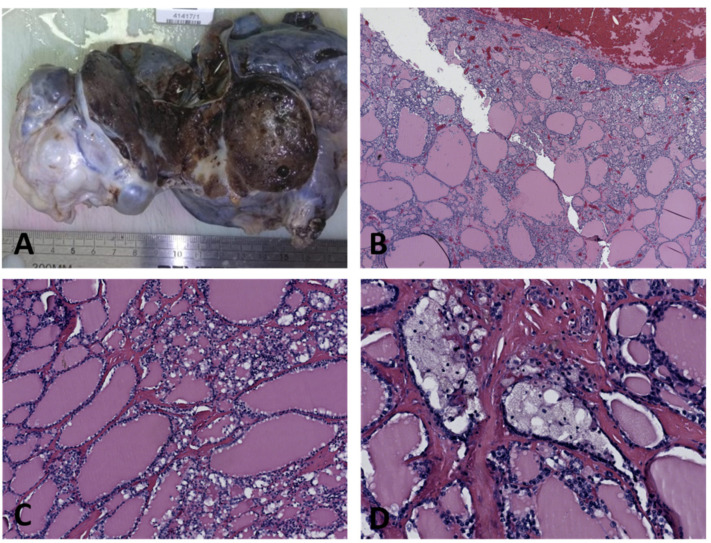
Struma ovarii in a 67-year-old patient: (**A**) Large ovarian mass with composite appearance, including thyroid-like areas, multiple cystic areas with colloid like content, variably hemorrhagic; (**B**) Most of the lesion is composed of thyroid follicles of variable caliber, with massive zonal hemorrhage (HE, ×25 magnification); (**C**) Anisofollicular thyroid tissue (HE, ×100 magnification); (**D**) Thyroid follicles of variable caliber, containing colloid and macrophages (HE, ×200 magnification) (courtesy of Dr. M. Stoicea).

Papillary carcinoma is the most common malignancy in struma ovarii and has the same histologic appearance as its thyroid counterpart (Figure 17).

Immunohistochemistry of the tumors is positive for thyroid-specific markers such as thyroglobulin and TTF1 [29]. CK19 expression and the loss of CD56 can highlight malignancy in difficult cases (e.g., extensive scarring or fibrosis in large lesions with a pseudoinvasive appearance) [30].

### 2.7.3. Treatment

Unilateral oophorectomy is the treatment of choice for an SOT. In the vast majority of cases the SOT is benign (90–95%) and has a good prognosis (61,62). In cases of malignant SOTs with extraovarian spread, an additional thyroidectomy is recommended followed by adjuvant therapy with I¹³¹ [65,66].

All these tumors that are detected in young female with desire to procreate required efforts to offer fertility-sparing surgery in carefully selected cases [67,68].

## 3. Conclusions

Ovarian germ cell tumors are rare, but unlike the epithelial ovarian cancers they could be early detected and diagnosed. Clinical signs and symptoms are non-specific, except for abdominal distension in certain large tumors. USs are an important diagnostic tool for the gynecologist. The presence of a solid mass in a US examination of an adolescent or young female may raise the suspicion of an OMGCT. To collect more information about this tumor, CTs and MRIs should be recommended even if they are not specific. In certain types of tumors serum markers are helpful but not specific.

Pathology is a cornerstone in the diagnosis of these tumors. Due to the rarity of this pathology, it is necessary that the diagnosis be established by expert pathologists, and additional IHC staining be part of the protocol.

Cases of OMGCTs must be managed by multidisciplinary teams in specialized centers. These clearly improve the quality of care and prognostic.

Surgery should be tailored according to age, interest in preserving fertility, and the stage of the disease. It is recommended that surgery should not be as aggressive as in epithelial ovarian cancers. Unilateral salpingo-oophorectomy is considered the actual standard of surgical treatment for young patients in an early stage of the disease. Surgical biopsies of the normal macroscopic contralateral ovary is not recommended. In premenopausal/menopausal patients with an advanced stage of the disease, a total abdominal hysterectomy with a bilateral salpingo-oophorectomy and staging should be recommended.

Platinum-based combination chemotherapy (cisplatin and carboplatin) has a high efficiency as an adjuvant treatment in the early stages, making it possible to achieve a survival rate of 90–95% in stage IA of the disease. Chemotherapy is very effective, even in the case of incomplete excision of the locally advanced disease, and when applied 75–80% of the cases could have a long-term survival. Recurrence after CMT is an indicator of poor prognosis. Radiation therapy does not have a place in the treatment of OMGCTs.

A follow-up is mainly based, in some types of tumors, on certain serum markers (AFP, β-hCG, and LDH). A CT of the pelvis, abdomen, and thorax should be recommended when clinically indicated.

A national tumor registry and protocol for these rare diseases is highly needed.

## Figures and Tables

**Table 1:** Cellular classification of OMGCTs *.

Tumor Type	
Primitive germ cell tumors	Dysgerminoma, yolk sac tumor, embryonal carcinoma, polyembryoma, choriocarcinoma, mixed germ cell tumors
Bi-or triphasic teratoma	Immature teratoma, mature cystic teratoma with malignant transformation
Monodermal teratoma	Malignant struma ovarii, malignant carcinoid, other malignant lesions

* Tavassoli FA, Devilee P. Pathology and genetics of tumors of the breast and female genital organs. Lyon, France: International Agency for Research on Cancer, 2003.

**Table 2:** World Health Organization classification of OGCTs (2014).

WHO Classification of OGCTs * (2014)
Dysgerminoma
Yolk sac tumor
Embryonal carcinoma
Non-gestational choriocarcinoma
Mature teratoma
Immature teratoma
Mixed germ cell tumor

* Prat J, Cao D, Carinelli S et al. Teratoma (Chapter 1: Tumors of the ovary). In Kurman RJ, Carcangiu ML, Herrington CS, Young RH (eds), WHO Classification of Tumors of Female Reproductive Organs, 4th edition. IARC: Lyon 2014; 57–62.

**Table 3:** Immunohistochemical markers of OGCTs.

Sall4	OCT3/4	CD30	CD117	D2-40	AFP	Glypican-3
Dysgerminoma +	+	−	+	+	−	−
Yolk sac tumor +	−	−	−	−	+	+
Embryonal carcinoma +	+	+	−	−	+/−	−

+ present and − absent.

**Table 4:** Serum markers associated with OGCT.

Tumor Type	hCG	AFP	LDH
Dysgerminoma	+/−	−	+
Endodermal sinus tumor (Yolk sac tumor)	−	+	+/−
Immature teratoma	−	+/−	+/−
Embryonal carcinoma	+	+	+/−
Choriocarcinoma	+	−	+/−
Mixed germ cell tumor	+	+	+

Legend: hCG—beta chorionic gonadotropin; AFP—alpha-fetoprotein; LDH—lactate dehydrogenase. (+ elevated and − normal)

**Table 5:** Differential immunohistochemical diagnoses of dysgerminoma *.

	*PLAP*	*CD117*	*OCT-4*	*CK*	*CD30*	*S100*	*LCA*	*MPO*
***Dysgerminoma***	+	+	+	−	−	−	−	−
***Embryonal carcinoma***	+	−	+	+	+	−	−	−
***Yolk sac tumor***	+	−	−	+	−	−	−	−
***Lymphoma***	−	−	−	−	−	−	+	−
***Granulocytic sarcoma***	−	+	−	−	−	−	+	+

* Dabbs D. Diagnostic Immunohistochemistry. Elsevier 2010. (+ present and − absent)

**Table 6:** Histological grading of an immature teratoma *.

Grade	Proportion of Immature Neuroectodermal Tissue Present on a Single Slide
1	≤LPF
2	2–3 LPF
3	≥4 LPF

* DiSaia, P.J., Saltz, A., Kagan, A.R., C.P. Morrow. Chemotherapeutic retro-conversion of immature teratoma of the ovary. Obstet Gynecol 1977;49(3):346–50. Legend: LPF—Low-power field.
